# Plant-Derived Inhibitors of AHL-Mediated Quorum Sensing in Bacteria: Modes of Action

**DOI:** 10.3390/ijms20225588

**Published:** 2019-11-08

**Authors:** Dmitry Deryabin, Anna Galadzhieva, Dianna Kosyan, Galimjan Duskaev

**Affiliations:** Federal Scientific Center of Biological Systems and Agrotechnologies of RAS, Orenburg 460000, Russia; annatolmacheva56@gmail.com (A.G.); kosyan.diana@mail.ru (D.K.); gduskaev@mail.ru (G.D.)

**Keywords:** quorum sensing (QS), AHL-mediated QS, bacterial virulence, biofilm, natural compounds, phytochemicals, quorum sensing inhibitors

## Abstract

Numerous gram-negative phytopathogenic and zoopathogenic bacteria utilise acylated homoserine lactone (AHL) in communication systems, referred to as quorum sensing (QS), for induction of virulence factors and biofilm development. This phenomenon positions AHL-mediated QS as an attractive target for anti-infective therapy. This review focused on the most significant groups of plant-derived QS inhibitors and well-studied individual compounds for which in silico, in vitro and in vivo studies provide substantial knowledge about their modes of anti-QS activity. The current data about sulfur-containing compounds, monoterpenes and monoterpenoids, phenylpropanoids, benzoic acid derivatives, diarylheptanoids, coumarins, flavonoids and tannins were summarized; their plant sources, anti-QS effects and bioactivity mechanisms have also been summarized and discussed. Three variants of plant-derived molecules anti-QS strategies are proposed: (i) specific, via binding with LuxI-type AHL synthases and/or LuxR-type AHL receptor proteins, which have been shown for terpenes (carvacrol and l-carvone), phenylpropanoids (cinnamaldehyde and eugenol), flavonoid quercetin and ellagitannins; (ii) non-specific, by affecting the QS-related intracellular regulatory pathways by lowering regulatory small RNA expression (sulphur-containing compounds ajoene and iberin) or c-di-GMP metabolism reduction (coumarin); and (iii) indirect, via alteration of metabolic pathways involved in QS-dependent processes (vanillic acid and curcumin).

## 1. Introduction

Quorum sensing (QS) is a cell–cell communication system that is ubiquitously used in microbial communities to monitor their population density and adapt to external environment. Firstly, QS was named and discovered in the marine bacterium *Vibrio fischeri* (now *Aliivibrio fischeri*) [1], where it regulates bioluminescence development in symbiotic “light” organs of squids from the genera *Euprymna* and *Sepiola*. This phenomenon involves LuxI synthase, which produces small diffusible signal molecules—acylated homoserine lactones (AHLs)—that accumulate in the environment. Upon reaching a high concentration, AHLs bind with LuxR activator protein and induce *lux*-operon transcription in a synchronous manner. Many other Gram-negative proteobacteria that belong to α, β and γ subdivisions use similar LuxI-type synthases and LuxR-type activator proteins. They utilise AHL-dependent gene expression mechanisms to perform processes that are not effective at low cell density but very useful for the microbial community at high cell density [2].

Notably, the virulence factors (toxins, proteases and immune-evasion factors) in many zoopathogenic and phytopathogenic bacteria, including *Pseudomonas* spp., *Acinetobacter* spp., and *Burkholderia* spp., are reportedly mediated by AHL. This fact positions QS as an attractive novel target for anti-infective therapy [3]. Another QS-related process is biofilm formation, in which bacterial cells attach to surfaces and envelop themselves in a secreted exopolymeric matrix. In contrast to bioluminescence, virulence factor biosynthesis and some other features, biofilm formation is not strictly switched on by AHLs. However, these phenomena are evolutionarily related [4], and some mechanisms of matrix development are under QS control [5]. Because QS interference aims to reduce virulence and inhibit biofilms but not necessarily kill bacteria, it probably does not exert selective pressure and is less likely to select for resistant strains compared to using conventional antibiotics.

Despite that the current list of cell-to-cell communication systems has significantly expanded, a variety of novel autoinducers have been identified, and that hierarchical or parallel QS networks that integrate several regulatory signals and receptors have been described [6], AHL-mediated systems remain the most attractive target for antivirulence therapy in several Gram-negative bacterial families [7]. Over the past 20 years, numerous artificial strategies have been proposed to combat AHL-mediated QS, including suppressing LuxI-type synthases, autoinducer degradation by enzymes (such as lactonases and acylases) or their sorption and sequestration in the environment, LuxR-type receptor antagonism and suppression of QS-activated genes [8]. However, the biopharmaceutical perspectives of these methods are still not completely understood.

An alternative approach is the search for natural compounds that show anti-QS activity. In particular, because higher plants co-evolved with the microbial environment and are constantly exposed to bacterial infections, it is logical to expect that these organisms developed have sophisticated chemical mechanisms to combat pathogens, including QS suppression [9].

The aim of this review was to summarize current data about the most significant groups of plant-derived inhibitors of AHL-mediated QS in bacteria with focus on the well-studied individual compounds which in silico, in vitro and in vivo studies taken together allow us to obtain the most complete knowledge about their modes of anti-QS activity.

## 2. Methodology for the Search and Study of Plant-Derived QS Inhibitors

The first step for screening of anti-QS activity is based on analyses of medicinal plants ethnobotanical descriptions. These species are known for their use in the treatment and prevention of bacterial infections in traditional medical practice [10]. Other higher plants that are potential natural QS inhibitor sources are some vegetables, fruits, berries, grains and spices [11]. These species are part of the human diet and may prevent the colonisation and invasion of bacterial pathogens.

The selected plant material is dried and treated with water, ethanol or ethyl acetate, which allows the most complete extraction of chemical compounds with different degrees of polarity [12]. The preliminary screening of the obtained extracts includes determination of their direct antibacterial effects, including the use of agar diffusion or micro-broth dilution assays [12,13,14]. For further studies, concentrations (dilutions) lower than the minimal inhibitory concentration (sub-MIC) only are used [14,15].

The second stage is aimed at screening plant extracts to determine biological activity against bacterial species that use AHL-mediated QS mechanisms for functional differentiation and biofilm formation. Apply the same methods as in the preliminary stage: diffusion of plant extract into agar; followed by measuring the area of suppression of pigments, the production of which depends on QS (any IQS activity is evident by the formation of a colourless, opaque, but visible halo around the well, due to a loss of pigmentation [12,13,14]); and the method of microbulion dilution (pigment is determined quantitatively by measuring the optical density using a spectrophotometer [12,13,15]).

The first method is qualitative and semi-quantitative, it allows the identification of QS inhibitors among plant extracts and to determine the preliminary degree of activity for the subsequent selection of concentrations to work with the dilution method, which is quantitative and allows the ranking of plant extracts by activity.

Two types of bioassays can be used for these studies. The first is based on AHL biosensors that have a functional LuxR-type protein but lack the LuxI-type synthase. The most popular biosensor is *Chromobacterium violaceum* 026 (NCTC 13278), a double mini-Tn5 mutant with insertion of this transposon in the *cviI* (*luxI*-type) gene, which synthesises the violet pigment violacein in response to C6-AHL autoinducer concentrations [13]. Other AHL biosensors are recombinant bacteria that carry plasmids with a LuxR-type protein-encoding gene and a QS-controlled promoter fused to the “reporter” genes, including *lux*- or *gfp*-operons [16,17]. The promoter activity in these strains depends on the presence of exogenous AHL. Thus, on one hand, the violacein production and bioluminescence level quantify the QS autoinducer presence in the environment; on the other hand, these bioassays allows the evaluation of the anti-QS activity of plant extracts at controlled AHL concentrations that induce violacein biosynthesis and bioluminescence development. Notably, these assays do not implicate the anti-QS properties that target AHL synthesis that may be studied using wild-type sensor strains. One example of an AHL-producing bacterium that is often used for anti-QS activity evaluation is C. *violaceum* ATCC 31532 [13], which synthesises C6-AHL and represents the initial strain for *C. violaceum* 026. Another example is *Pseudomonas aeruginosa* PAO1 (ATCC 15692) [18], which exploits the hierarchical network, including two AHL molecules—3-oxo-C12-AHL and C4-AHL—that bind to LasR and RHLR transcriptional regulators, respectively. In these assays, screening methods for anti-QS activity include the quantification of AHL-regulated violacein biosynthesis in *C. violaceum* or virulence traits (e.g., pyocyanin production) in *P. aeruginosa*, as well as biofilm formation by crystal violet staining or confocal laser scanning microscopy. The obtained results show the typical presence of anti-QS properties in numerous plants that grow in the Eastern [12] and Western [14] Hemispheres, as summarised in Koh et al. [19]. These findings provided the basis for continuing the study of this bioactivity.

At the next stage, the effects of plant extracts with proven anti-QS properties are analysed on individual chemical compounds to demonstrate this bioactivity. In some cases, it is possible to isolate these compounds by direct plant extract fractionation, for example, using high-performance liquid chromatography followed by confirmation of their structure via mass spectrometry and nuclear magnetic resonance spectroscopy [20]. In other cases, the reverse phase high performance liquid chromatography can lead to dissipation and loss of anti-QS activity detected in the total extract and an excellent resolution of a majority of the compounds may be done with the gas chromatography–mass spectrometry [20] or similar chemical analytical techniques. Subsequently, natural or chemically synthesised analogs of the identified plant-derived molecules are tested for anti-QS and anti-biofilm properties as described above. These tests are used to determine whether the bioactivity of the plant extracts is due to the presence of one or more bioactive compounds.

Additionally, some in vivo models (from protozoa to vertebrates) are used to prove antivirulence activity of the identified QS inhibitors. The invertebrate *Caenorhabditis elegans* model is very valuable for this endeavour. It allows high-throughput screening and provides deep insight into QS modulation, virulence regulation and, in general, the potential of novel anti-infectives [21]. Mammalian models, such as rats or mice, have immune systems and general physiology that closely resemble humans and are also common models used to investigate QS inhibitor impacts on bacterial infections [22]. The same in vivo models allow the evaluation of the toxicity of selected plant molecules [23], an important factor for their potential pharmacological use.

The biosynthetic classes of anti-QS compounds, their biological activity in in vitro and in vivo assays and the in which plant families they occur are summarised in a valuable review by Ta and Aranson [24].

The current stage of plant-derived molecule research aims to identify their targets and mechanisms that effectively inhibit QS development in bacterial populations.

The in silico approach, which is used in medicinal chemistry for the discovery of enzyme inhibitors, is often applied to discover anti-QS compounds and evaluation of their bioactivity mechanisms. This approach involves combining several computational methods, including molecular docking with multiple conformations and molecular dynamic simulations, in order to estimate binding affinity of the investigated plant-derived molecules to QS-system-related proteins [25]. In some cases, the interaction between plant-derived molecules and target proteins is confirmed by direct protein–ligand interaction studies [26]. A limitation of this approach is the small number of analysed targets (usually LuxR-type and LuxI-type proteins); this constraint does not allow evaluation of the complex effect of plant-derived molecules on the full range of possible target proteins.

Another popular method for QS research is proteomic analysis, which includes initial quantitative 2D-difference gel electrophoresis followed by identification of proteins in each spot by mass spectrometry or nuclear magnetic resonance spectroscopy. This approach is very useful for the examination of a range of proteomic and metabolomic changes in wild-type cultures and QS mutants, as well as to determine the impact of various factors on the QS-controlled changes in wide proteins spectra in model bacteria [27].

A better understanding about QS modulation in response to plant-derived molecules can also be obtained by transcriptome analysis. This analysis includes total RNA extraction of treated and untreated bacterial samples, RNA reverse transcription into a complementary DNA (cDNA) library and subsequent comparative analysis by various genetic techniques. Early (and some current) studies use(d) real-time polymerase chain reaction (RT-qPCR), but this approach is limited by the number of analysed genes, which typically include QS regulatory and QS-regulated virulence genes [28]. More informative approaches are based on high-throughput sequencing methods that allow quantitative study of complete transcription profiles using RNA-seq [29] or high-density oligonucleotide microarrays [30]. In turn, the subsequent bioinformatics analyses reveal differentially expressed (up- and downregulated) genes, allow their functional annotation in a variety of biological processes and finally characterise global gene expression patterns in response to QS inhibitors.

## 3. Plant-Derived Molecules that Affect AHL-Mediated QS in Bacteria

### 3.1. Sulphur-Containing Compounds

Specialised sulphur-rich metabolites are characteristic for allium vegetables, such as garlic, onion, leeks, and cruciferous vegetables, including cabbages, kales and broccoli [31]. In macerated plant tissues, the sulphur-containing precursors (for example, *S*-alk(en)yl cysteine sulphoxide alliin in *Allium sativum*) enter the cleavage cascade initiated by C–S lyases (i.e., alliinases) [32]. Another mechanism is via the myrosinase-assisted hydrolytic cleavage of glucosinolates that leads to isothiocyanate production. The secondary metabolites that are formed by this catalytic breakdown are further transformed into thiosulphinates. These compounds include a variety of organosulphur compounds, including allicin and ajoene detected in allium vegetables and isothiocyanates from cruciferous vegetables extracts.

Previous research showed plant-derived sulphur-containing compounds have anti-QS bioactivity [24]. These actions include downregulation of QS-dependent virulence factors and biofilm development in *P. aeruginosa* by allicin and ajoene, QS inhibition in *C. violaceum* and reduction of biofilm formation in *P. aeruginosa* and *Listeria monocytogenes* by sulphoraphane and allyl isothiocyanate. A screening of a 25 disulphide-bond-containing compound library that structurally resemble ajoene revealed the two most active QS inhibitors in a bioreporter assay [33].

The current list of garlic bioactive sulphur-containing compounds includes diallyl disulphide, which can inhibit *P. aeruginosa* virulence factors at sub-MIC concentrations by inactivating the transcription of key genes across three different QS systems [34]. In particular, diallyl disulphide decreases *lasR* (encodes a regulator protein) transcription that further downregulates the transcription of *phzM* (encodes pyocyanin), *pslB* (responsible for the production of a biofilm matrix polysaccharide) and *chiC* (encodes chitinase).

Anti-QS compound screening in cruciferous vegetables identified the previously known sulphoraphene as well as the natural isothiocyanate erucin from broccoli [35]. Evaluation of these effects suggests that isothiocyanates are antagonists of the transcriptional activator LasR that induces numerous QS-dependent virulence factors in *P. aeruginosa*. In another study, the chromatographic separation of horseradish extract led to the isolation of a sulphur-rich anti-QS compound that was identified by liquid chromatography-diode array detector-mass spectrometry and nuclear magnetic resonance spectroscopy as the isothiocyanate iberin [36]. In this study, iberin dose-dependently affects *lasB-gfp*, *rhlA-gfp*, *rhlR rhlA-lacZ* and *luxR*-P*luxI-gfp* sensor strains and inhibits rhamnolipid production and biofilm formation in *P. aeruginosa*.

A comparative systems biology approach that combined the use of transcriptomic and proteomic analyses determined the iberin bioactivity mechanism on *P. aeruginosa* [37]. RNA-seq-based transcriptomics demonstrated the inhibition of GacA-dependent small regulatory RNA (sRNA) *RsmY* and *RsmZ*, while quantitation proteomics showed that iberin reduces the abundance of the LadS protein, an activator of GacS. The same mode of action was revealed for ajoene: this sulphur-rich compound lowers expression of the sRNAs *RsmY* and *RsmZ* in *P. aeruginosa* [38]. Taken together, these findings suggest that a broad range of plant-derived organosulphur compounds affect QS through downregulation of the Gac/Rsm network and lowers the expression of regulatory sRNAs that consequently increases free RsmA RNA-binding protein. These alterations modify numerous phenotypic traits, including biofilm formation and QS expression (Table 1).

### 3.2. Monoterpenes and Monoterpenoids

Monoterpenes are members of a large and diverse class of hydrocarbon organic compounds, named “terpenes” (from an obsolete form of the word “turpentine”), produced by a variety aromatic plants, including some *Monocots* and *Eudicots* species. Recent plant genome sequencing showed at least 17 species that contain the terpene synthase gene. Additional enzymes are required for terpene biosynthesis and their scaffold modification [70] in the cell cytoplasm by the mevalonic acid pathway or in plastids by the MEP-(2-methyl-D-erythritol-4-phosphate) pathway. Monoterpenes have the common formula C_10_H_16_, consist of two isoprene units, may have a linear (linalool, myrcene) or cyclic (*limonene*, eucalyptol, terpinene, pinene, et cetera) structure and are the primary constituents of many plant-derived essential oils.

In Luis et al. [71], the monoterpene-rich oils from *Rosmarinus officinalis (contains limonene), Eucalyptus citriodora (contains* eucalyptol) *and Lavandula angustifolia* (contains linalool) inhibit the *C. violaceum QS mechanisms*. In another study, eucalyptol-rich essential oil from *Eucalyptus globulus and* limonene-rich essential oil from *Eucalyptus radiata* inhibit AHL-mediated violacein pigment production in *C. violaceum* without interfering with bacterial growth [72]. Mandarin essential oils contain high monoterpene quantities, mainly limonene, γ-terpinene, myrcene and α-pinene in sub-MIC concentrations. They significantly reduce AHL production in *P. aeruginosa* and ultimately reduce QS-dependent elastase activity [73].

The structurally related compounds (monoterpenoid phenols or terpenoids) thymol and carvacrol extracted from Thymus vulgaris L. and other *Lamiaceae* species are discussed in a review [24] and an original article [39] as predominantly anti-biofilm compounds. *Carum copticum* essential oil, with high contents of thymol, p-cymene, γ-terpinene and β-pinene, shows notable anti-QS activity in *C. violaceum* [74]. Comparable sub-MIC carvacrol concentrations also reduce both *cviI* expression in *C. violaceum* and inhibit QS-dependent violacein biosynthesis and chitinase activity [40] as well as QS-controlled pyocyanin production in *P. aeruginosa* [41]. The same effects were confirmed against plant pathogens *Pectobacterium aroidearum* and *Pectobacterium carotovorum* subsp. *Brasiliense*, where carvacrol suppresses production of QS signalling molecules and the expression of genes strictly controlled by QS [42]. The modelling data suggest that carvacrol can directly interact with both homoserine lactone synthase (ExpI) and transcriptional regulator (ExpR) with good stereochemical qualities. Indeed, 98.7% (ExpR) and 98.9% (ExpI) of residues occur in the most favoured conformation and are in the allowed regions of the Ramachandran plot. In turn, the docking scores of carvacrol support its potential binding to ExpI/ExpR, with stronger interactions than previously known for another QS inhibitor, namely, halogenated furanone C30 [42].

Li et al. presented consistent data about terpenoids bioactivity mechanisms [43]. They focused on the l-carvone (most abundant in the essential oils from seeds of *Carum carvi* and *Mentha spicata*) effects against the QS system in the opportunistic pathogen *Hafnia alvei*. The experiments included RT-qPCR and demonstrated reduction in AHL production and swinging/swarming motility. Furthermore, in silico analysis of homoserine lactone synthase HalI and transcriptional regulator HalR revealed they have a higher affinity for l-carvone compared to halogenated furanone C30.

Taken together, the present data characterise monoterpenoids as bifunctional molecules that reduce AHL biosynthesis by interaction with LuxI-type proteins and interfere with AHL reception through binding with LuxR-type proteins (Table 1).

### 3.3. Phenylpropanoids

Phenylpropanoids are a diverse family of organic compounds that contain a six-carbon aromatic phenyl group and a three-carbon propene tail. In plants, these compounds are synthesised by the shikimate pathway from phenylalanine or tyrosine amino acids [75] and are then involved in numerous secondary biosynthetic pathways.

The most studied compound of this group is cinnamaldehyde (occurs naturally predominantly as the trans E isomer called trans-cinnamaldehyde). It was first isolated from cinnamon essential oil, but it also occurs in cassia, patchouli and hyacinth essential oils. Sub-inhibitory cinnamaldehyde concentrations significantly inhibit expression of QS-dependent virulence genes and biofilm formation in *P. aeruginosa* without detectable bactericidal effect [28]. Transcriptome analysis demonstrated effective down-regulation of both the *las* and *rhl* QS systems that reduce production of extracellular virulence factors: protease, elastase and pyocyanin. In other study the cinnamaldehyde was effective against QS-controlled extracellular protease, swimming and swarming motility, and biofilm formation in *Pseudomonas fluorescens* [44]. Using the GC–MS technique, it was shown that the cinnamaldehyde did not interfere with AHL production, whereas molecular docking analysis revealed that this plant-derived compound can interact with the LuxR-type protein of *P. fluorescens*. Similar in structure, trans-anethole (main component of *Pimpinella anisum* anise oil) confirmed the phenylpropenoids anti-QS potential and showed inhibition of QS-regulated virulence factors in *P. aeruginosa* while molecular docking and protein-ligand interaction studies suggested that trans-anethole binds to the LasR regulatory protein [26].

Chang et al. presented an opposite view on phenylpropanoids bioactivity [44]. In that study, cinnamaldehyde efficiently inhibits AHL production in *P. aeruginosa*, while molecular docking analysis suggests that cinnamaldehyde binds to LasI synthase, interacts with LasI Phe27 and Trp33 and forms a hydrogen bond with the Arg30 residue. This residue forms the substrate-binding pocket and is completely conserved in the LuxI synthase family. Notably, despite direct LuxR-type and LuxI-type protein binding properties, cinnamaldehyde and substituted cinnamaldehydes show pleiotropic effects on QS systems, including their ability to interfere with AI-2-mediated communication [45]. In that study, the active compounds did not reduce AI-2 production, but the regulator protein DNA-binding ability is decreased.

Another well-studied phenylpropene-related plant compound is eugenol, named from the former Linnean nomenclature term for cloves (*Eugenia caryophyllata*, currently *Syzygium aromaticum*). It was further extracted from certain essential oils, including nutmeg, cinnamon, basil and bay leaf. This compound was identified during the screening of herbal and clove extracts and can inhibit QS-controlled gene expression in recombinant *Escherichia coli*, *P. aeruginosa* QSIS-*lasI* and *C. violaceum* CV026 biosensors [46]. Eugenol examination against the standard *P. aeruginosa* strain and multi-drug resistant clinical isolates confirmed the inhibition of elastase, protease, pyocyanin and pyoverdine biosynthesis as well as extracellular polysaccharides and rhamnolipid production. Further, in silico docking studies demonstrate stable molecular binding between eugenol and QS receptor LasR. This data suggests that this mechanism may lead to significant repression of QS-controlled genes in *P. aeruginosa* [47].

Recently, Lou et al. [48] confirmed the inhibitory effect of eugenol and its nanoemulsion on QS-controlled virulence factors and biofilm formation in *P. aeruginosa*. Additionally, these compounds downregulate the expression of the QS synthase genes *lasI* and *rhlI*, changes that lead to reduced production of both 3-oxo-C12-AHL and C4-AHL signal molecules. In Joshi et al. [42], eugenol also reduces the production of AHL, expression of QS-related genes, biofilm formation and activity of plant-cell-wall-degrading enzymes in the phytopathogens *Pectobacterium carotovorum* subsp. *brasiliense* and *Pectobacterium aroidearum*. Based on docking computational models, the mechanism of action of these anti-QS compounds probably involves direct interaction with both ExpI (acyl-HSL synthase) and ExpR (regulatory protein).

To better understand eugenol modes of action, transcriptome sequencing in *Klebsiella pneumoniae* was performed [49]. There is a total of 5779 differentially expressed genes enriched in a variety of biological processes and pathways. To better understand eugenol modes of action, transcriptome sequencing in *Klebsiella pneumoniae* was performed [76] where a total of 5779 differentially expressed genes enriched in a variety of biological processes and pathways were identified. The transcriptional data showed that exposure to eugenol involved a series of gene ontologies in biological processes, cellular components and molecular functions. Eugenol can reduce AI-2 generation by downregulating synthesis of *luxS* and *lsrK* as well as increasing the expression of the repressive gene *lsrR*. Thus, although *K. pneumoniae* uses the AI-2-based QS system, the transcriptome sequencing showed pleiotropic eugenol effects.

In sum, this data indicates that eugenol and other phenylpropanoids not only directly interact with LuxI-type and LuxR-type proteins but are involved in numerous QS-related processes (Table 1).

### 3.4. Benzoic acid Derivatives

Benzoic acid is a simple aromatic carboxylic acid named from “gum benzoin” (a balsamic resin from the bark of several species of trees in the genus *Styrax* that contain up to 20% benzoic acid and 40% benzoic acid esters). Currently, benzoic acid occurs naturally in many plant species [76]. It is produced from cinnamic acid and an intermediate in the biosynthesis of many other secondary metabolites. Appreciable amounts of benzoic acid are found in most berries of several Vaccinium species, e.g., cranberry (*Vaccinium vitis macrocarpon*) and bilberry (*Vaccinium myrtillus*).

The representative data obtained for benzoic acid derivatives are for vanillin and vanillic acid. In Ponnusamy et al.’s study [77], vanillin from the extract of vanilla beans (*Vanilla planifolia* Andrews) inhibits QS-dependent violacein biosynthesis in *C. violaceum* and biofilm formation in *Aeromonas hydrophila*. Additionally, this compound significantly silences short-chain C4-AHL and long-chain 3-oxo-C8-AHL autoinducer production. The inhibitory characteristics of vanillin as an anti-QS agent against biofilm formation was confirmed in several studies that use membrane-based applications [50].

Vanillic acid from kiwi (*Actinidia deliciosa*) pulp extract also significantly affects QS-regulated virulence and biofilm formation in a *Serratia marcescens* clinical isolate and increases the survival of *Caenorhabditis elegans* upon *S. marcescens* infection [78]. To evaluate these anti-QS effects, proteomic analysis using 2-dementional electrophoresis with mass spectrometric identification of expressed proteins in bacterial cells cultured in the presence and absence of vanillic acid was performed. Based on the densitometric analysis, among the 579 detected spots, 27 spots were downregulated and 21 were upregulated. Gene ontology analysis of differentially expressed proteins revealed significant changes in proteins involved in S-layers, protease, prodigiosin and lipase production as well as on biosynthesis of flagella, amino acids, carbohydrates and lipids in *S. marcescens*. Surprisingly, there is no evidence of vanillic acid activity on autoinductor synthases and receptors. The data suggest that this compound exhibits unusual anti-QS effect that is probably mediated through impaired fatty acid biosynthesis (Table 1).

Some other benzoic acid derivatives are also anti-QS and anti-biofilm agents. Gallic acid [24] inhibits *P. aeruginosa* and *Eikenella corrodens* biofilm formation, and, in terms of QS, reduces violacein production in the *C. violaceum* bioassay. Similar bioactivity was recently shown for methyl gallate [51], which profoundly modulates biofilm, motility, proteolytic, elastase, pyocyanin and rhamnolipid biosynthesis, as well as dose-dependent suppresses of *lasI*/*R*, *rhlI*/*R* and *pqsA* gene expression in *P. aeruginosa*. Additionally, methyl gallate suppresses both AHL synthesis and activity in the *C. violaceum* bioassay. However, detailed proteomic and/or transcriptomic analyses for gallic acid and methyl gallate bioactivity mechanisms evaluation are not yet reported.

### 3.5. Diarylheptanoids

Diarylheptanoids are a relatively small class of plant secondary metabolites that consist of two aromatic rings joined by a seven-carbon chain and varying in some substitutions [79]. According the chemical structure, they are further subdivided into linear (the most numerous subgroup is named “curcuminoids”) and cyclic diarylheptanoids. They are present in plants from 10 different families, e.g., *Betulaceae* and *Zingiberaceae*, where the best-known compound is curcumin isolated from the rhizome of *Curcuma longa*.

Curcumin inhibits violacein biosynthesis in *C. violaceum* as well as virulence factor production in *Vibrio* sp., *S. marcescens* and *P. aeruginosa* [24]. In Rudrappa and Bais’ study [80] this compound effectively inhibited biofilm formation, pyocyanin biosynthesis, elastase/protease activity, and AHL production in *P. aeruginosa* and as a consequence of this, reduced its pathogenicity in plant (*Arabidopsis thaliana*) and animal (*Caenorhabditis elegans*) infection models [80].

The list of diarylheptanoids that exhibit anti-QS activity has recently expanded to include platyphyllenone and hirsutenone from the barks of *Alnus viridis* ssp. *viridis* (green alder) and *Alnus glutinosa* (black alder), respectively [52]. Both compounds negatively influence biofilm formation, motility and pyocyanin biosynthesis and significantly decrease AHL production in *P. aeruginosa*, more specifically, the long chain C12-oxo-AHL. The anti-QS activity of *Amomum tsaoko* (*Amomum tsao-ko Crevostet Lemarie*) on *C. violaceum* and some foodborne pathogens [81] is also mediated by another diarylheptanoid [7-(4-hydroxyl-3-methoxyphenyl)-1-(4 hydroxyphenyl)-hepta-4E,6E-dien-3-one] that is a major component of this plant extract.

To elucidate the diarylheptanoid anti-QS mechanism, molecular docking analysis of curcumin with active sites of LuxI-type synthase and LuxR-type receptor protein of opportunistic pathogen *Aeromonas sobria* was performed [81]. The in silico analysis showed a higher binding affinity of curcumin to the LuxI-type protein. This compound interacts with key residues (VAL143, ARG102, LEU103, SER145 and ILE105) via hydrogen bonds. Gas chromatography-mass spectrometry analysis showed that free curcumin and curcumin liposome treatment of *A. sobria* decreases the production of C4-AHL, C6-AHL, C10-AHL and C14-AHL. This data confirms the ability of curcumin to block LuxI-type synthases. Further, Bihari et al. [82] demonstrated that *P. aeruginosa* treated with sub-MIC curcumin reduces C12-oxo-AHL and C4-AHL signal molecules production, while expression of QS regulatory genes *lasI*, *lasR*, *rhlI* and *rhlR* in presence of this compound is significantly decreased compared with untreated *P. aeruginosa* probe.

Proteomic, mass spectrometric and gene ontology analysis were employed to unearth the underlying molecular mechanism responsible for the anti-QS activity of curcumin [53]. 

Surprisingly, the obtained data reveal curcumin predominantly as a regulator of iron acquisition, iron storage and detoxification of reactive oxygen species.

In vitro assays also confirmed the alterations in catalase, superoxide dismutase and sensitivity of *P. aeruginosa* to H_2_O_2_ upon curcumin treatment as well as inhibition of QS-regulated pyocyanin and pyoverdine pigments. These results suggest curcumin bioactivity can attenuate QS by targeting iron homeostasis, oxidative stress response and the biosynthesis of metabolic intermediates involved in virulence factors production (Table 1).

### 3.6. Coumarins

Сoumarins (1,2-benzopyrones or 2H-1-benzopyran-2-ones) are a benzopyrone class of organic compounds. Structurally, they are constructed from a benzene ring fused to α-pyrone ring [54]. Their name comes from a French term for the tonka bean (*Dipteryx odorata*), from which simple coumarin was first isolated in 1820. In high plants, these compounds are biosynthesised from phenylalanine via the shikimic acid pathway. They are currently identified as secondary metabolites in approximately 150 different species distributed over nearly 30 families, of which a few important ones are *Rutaceae, Umbelliferae, Clusiaceae, Guttiferae, Caprifoliaceae, Oleaceae, Nyctaginaceae* and *Apiaceae* [55]. Except for a few rare cases, plant-derived simple coumarins (including scopoletin, aesculetin and umbelliferone) contain hydroxyl- or methoxy- substitutions in the benzene ring and often occur as glycosides. Other closely related coumarin derivatives include furocoumarins and isofurocoumarins, as well as various pyranocoumarins, biscoumarins and dihydroisocoumarins.

Coumarins possess both QS and biofilm inhibitory activities. Aesculetin and umbelliferone inhibit QS in *C. violaceum* and *P. aeruginosa*, lower the expression of biofilm-related genes and reduce virulence in a *C. elegans* infection model [24]. Similar activity is suggested for naturally occurring furocoumarins from grapefruit (*Psoralea corylifolia* L.) juice and extract [83]. These coumarin-related compounds (bergamottin and dihydroxybergamottin) inhibit both AHL- and AI-2-mediated signalling in a *Vibrio harveyi* bioassay. They also reduce QS-mediated swarming motility and biofilm formation in *P. aeruginosa*. Further anti-QS activity was confirmed for coumarin itself [84]. Using a range of *S. marcescens* SP15, *C. violaceum* DSM 30191 and *A. tumefaciens* NTL4 biosensor strains, coumarin is effective against short-, medium- and long-chain AHL-induced QS. In *P. aeruginosa*, coumarin also inhibits biofilm formation, phenazine production and swarming motility, potentially linked to reduced expression of the *rhl* and *pqs* QS genes.

To test the significance of substitutions on the coumarin framework for anti-QS activity, D’Almeida et al. [56] compared seven structurally related compounds (simple coumarin and its different hydroxylated derivatives). Using the mutant *C. violaceum* CV026 biosensor strain, the authors demonstrated that all tested coumarins, with the exception of 4-hydroxycoumarin and dihydrocoumarin, inhibit AHL-mediated violacein production. In a follow-up test using the wild-type *C. violaceum* ATCC 12472 strain, both 4-hydroxycoumarin and dihydrocoumarin reduce the percentage of violacein production, although to a lesser extent compared to the other tested coumarins. Additionally, the coumarins aesculetin and umbelliferone show the highest biofilm inhibition in *P. aeruginosa*, while 4-hydroxycorumarin and dihydrocoumarin presents the lowest bioactivity.

The initial ideas about the coumarin anti-QS mechanism were based on a virtual screening of a traditional Chinese medicine library by docking analysis against the *Agrobacterium tumefaciens* QS transcriptional activator protein TraR [57]. This study revealed that the simple coumarin aesculetin (6,7-dihydroxycoumarin) and coumarin glucoside aesculin are structurally compatible with the TraR AHL-binding site.

However, the current data indicate another mode of coumarin activity against QS-controlled features in bacteria. In Zhang et al. [85], transcriptome analysis revealed numerous genes involved in the *las, rhl*, PQS and integrated QS network are downregulated in coumarin-treated *P. aeruginosa* PAO1 cells. Furthermore, the expression of genes related to type III secretion and cyclic diguanylate (c-di-GMP) metabolism are also significantly reduced. c-di-GMP is a low-molecular-weight intracellular secondary messenger that controls transcription and upregulates535 genes and downregulates 432 genes in *P. aeruginosa* cells with low c-di-GMP content compared to cells with high c-di-GMP content [58]. Thus, reduced c-di-GMP metabolism may be the cause of QS operon repression that leads to virulence factors inhibition, decreasing motility and enhanced biofilm formation in *P. aeruginosa* cells treated with coumarins (Table 1). In turn, TpbA/TpbB proteins may be primary targets for coumarin, where TbpB is a suppressor of tyrosine phosphatase TpbA, which negatively regulates intracellular c-di-GMP concentrations [59].

### 3.7. Flavonoids

Flavonoids (named from the Latin word “*flavus*” meaning yellow color) are a class of diverse plant-derived secondary metabolites with variable phenolic structures [60]. Chemically, these molecules have a common 15-carbon skeleton that consists of two coupled phenyl and heterocyclic ring and a second phenyl ring; they are generally called A, C and B and abbreviated as C6-C3-C6. Depending on the carbon of the C ring on which the B ring is attached, these molecules can be classified as bioflavonoids (the B ring is linked in position 2 of the C ring), isoflavonoids (the B ring is linked in position 3 of the C ring) or neoflavonoids (the B ring is linked in position 4 of the C ring). In nature, these compounds are synthesised by the phenylpropanoid pathway [61], where the amino acid phenylalanine is used to initially produce 4-coumaroyl-CoA. Subsequently, numerous polyphenolics, tannins and flavonoids can be formed. Flavonoids are ubiquitous in plants, and over 5000 naturally occurring flavonoid-related compounds have been characterised. The highest flavonoid content occurs in parsley, onions, blueberries and other berries, bananas and all citrus fruits, among others.

The flavonoids quercetin, naringenin, sinensetin and apigenin from citrus extracts inhibit QS-regulated bioluminescence in *Vibrio harveyi*, whereas catechins from green tea (*Camellia sinensis* L.) decreased violacein and virulence factors production in *C. violaceum* and *P. aeruginosa*, respectively [61]. A kaempferol-rich extract from the medicinal herb *Centella asiatica* L. shows similar bioactivity; it completely inhibits violacein production in *C. violaceum* and dose-dependently represses the QS-regulated phenotype, namely, pyocyanin production, elastolytic and proteolytic activities, swarming motility and biofilm formation in *P. aeruginosa* [86]. The naringin-rich extract from pummelo peel (*Citrus maxima* (Burm.)) inhibits violacein production in *C. violaceum* as well as biofilm production and swimming motility in the biosensor strain *Vibrio anguillarum* [87]. Quercetin and quercetin-3-O-arabinoside anti-QS activity was confirmed during examination of flavonoid-rich from medicinal plant *Psidium guajava* L. [88]. Both extract and flavonoic compounds inhibit violacein production in *C. violaceum* and pyocyanin production, proteolytic, elastolytic activities, swarming motility and biofilm formation in *P. aeruginosa*. Interestingly, anti-QS activity of these compounds is not related to AHL synthesis inhibition: the supernatant of wild-type *C. violaceum* 31532 growing in presence of the flavonoid-rich fraction induces violacein production in the mutant *C. violaceum* CV026 sensor strain, the same as the control sample.

Optimised screening of 465 natural and synthetic flavones library examinations using wild and mutant AHL-deficient *C. violaceum* strains demonstrated that small side -OH or -OCH3 groups on the B-ring positively influence the QS inhibitor activity [89]. Another structure–activity analysis was performed during the screening of natural product derivatives library (*n* = 3040) [62]. Among the highly active compounds, two structurally similar flavonoid derivatives that affected all the studied QS-related functions at micromolar concentrations were identified. In these flavonoid molecules, the esters that differ in the side chain length carboxylic or fatty acid are linked to the flavone skeleton on the C-ring that indicate the importance of the hydrophobic interaction of such ligands with intracellular targets.

Gopu et al. evaluated flavonoid bioactivity mechanism. In silico techniques like molecular docking and molecular dynamics simulation revealed that quercetin binds more rigidly with *P. aeruginosa* transcriptional regulator LasR receptor protein than the signalling compound [90]. This analysis also predicted that the quercetin QS inhibitory activity occurs through conformational changes between the receptor and quercetin complex. Further in silico studies suggest that quercetin can act as a competitive inhibitor for QS signalling compound by placement in the AHL-binding pockets of the LasR protein of *P. aeruginosa* [91]. There is a better interaction and placement of quercetin aglycone in the structures of the *C. violaceum* transcriptional regulator CviR receptor protein of than the glycosylated compound quercetin 3-β-D-glucoside, findings that are consistent with the data about its better QS inhibitory effect.

Another view is presented in a study where flavonoids inhibited QS via antagonism of the autoinducer-binding LasR and RhlR receptors in *P. aeruginosa* [63]. Biochemical analyses revealed that these compounds function non-competitively to prevent LasR/RhlR DNA binding and are allosteric inhibitors. This finding was supported by combination of molecular docking, molecular dynamics simulations and machine learning techniques that showed two binding modes of quercetin with LasR protein [92] (Table 1). One is the non-competitive interaction with the ligand binding domain, and the second is the interaction with the LBD-SLR-DBD “bridge”, which involves conservative amino acid interactions and it is also non-competitive. In both studies, the structure–activity relationship analyses demonstrated that the presence of two hydroxyl moieties in the flavone A-ring backbone are essential for potent inhibition of LasR/RhlR [63], where hydroxyl group of ring A of quercetin is necessary for interaction with Leu177 in the LasR during the second binding mode [91].

### 3.8. Tannins

Tannins are a class of highly-polymerised polyphenolic secondary metabolites with molecular weights that range from 500 up to 20,000 Da. These compounds are named from Latin word “tannāre” (oak bark) that reflects to the use of oak in tanning animal hides and due to the ability of tannin’s hydroxyl groups to form strong complexes with various macromolecules, especially proteins [64]. According to the base unit of the monomer, tannins may be divided into two major groups: hydrolysable tannins, formed from gallic acid (such as ellaginannins), and condensed flavone-derived tannins (proanthocyanidins) [65]. Interestingly, these compounds are biosynthesised and polymerised in newly discovered organelles called “tannosomes” [93]. These organelles are vacuoles formed from the chloroplast membrane; they carry tannins to large vacuoles filled with acidic fluid where tannins are stored without interaction with cytoplasmic proteins. The most significant plant families that contain tannins are: *Najadaceae*, *Typhaceae* in monocot and *Aceraceae, Actinidiaceae, Anacardiaceae, Bixaceae, Burseraceae, Combretaceae, Dipterocarpaceae, Ericaceae, Grossulariaceae, Myricaceae* for dicot (where these compounds are found in leaf, bud, seed, root, and stem tissues). Hydrolysable tannins are typically present in plants from temperate woods, while tropical woods tend to contain condensed tannins.

Hydrolysable tannins have apparent diverse anti-QS properties. Adonizio et al. first examined ellagitannins (castalagin and vescalagin, which are 1,2,3,5-nonahydroxytriphenoyl-4,6-hexahydroxydiphenoyl-glucose isomers) [94,95] isolated from the south Florida medicinal plant *Conocarpus erectus* (*Combretaceae*). The tannin-rich extract and fractionated compounds significantly inhibit numerous QS-controlled factors in *P. aeruginosa* with marginal effects on bacterial growth as well as prevent *C. elegans* mortality in a *P. aeruginosa* gut infection model. Li et al. [66] showed that punicalagin (an ellagitannin found in pomegranate, *Combretaceae* and *Myrtales* species) inhibits violacein production in *C. violaceum* and downregulates QS- and motility-related genes in *Salmonella typhimurium*. Anti-QS activity of tannin-rich fraction from pomegranate rind was confirmed using the *C. violaceum* bioassay [67]. Further, transcriptional analysis showed that this fraction downregulates the expression of curli genes (*csgB* and *csgD*) and various motility genes (*fimA, fimH, flhD, motB, qseB*, and *qseC*) in *Escherichia coli*. Broad spectrum anti-QS activity of hydrolysable tannin-rich extracts of Indian medicinal plants *Phyllanthus emblica, Terminalia bellirica, Terminalia chebula, Punica granatum, Syzygium cumini* and *Mangifera indica* was reported by Shukla and Bhathena [96]. The authors suggest the tannin bioactivity mechanism is mediated by protein precipitation that may disrupt QS by inactivating various enzymes responsible for the autoinducer synthesis or by binding to protein receptors of QS signals.

Another study presented evidence of the activity of hydrolysable tannins against N-acyl homoserine lactone synthase [45]. Like two other molecules (salicylic acid and trans-cinnamaldehyde), tannic acid reduces AHL production in L-arabinose induced *E. coli* MG1655 [pBAD-rhlI], which carries functional RhlI synthase of *Pseudomonas aeruginosa*. However, if such activity of “small” molecules (in particular, cinnamaldehyde) can be explained by the occupation of the substrate-binding pocket on the AHL-synthase, the same bioactivity mechanism of a “large” tannic acid molecule remains unknown. A possible explanation for this fact is the ability of ellagitannins to be hydrolysed to ellagic acid and then metabolised to urolithin-A and urolithin-B. For example, the ellagic-acid-containing fraction from the *Terminalia chebula* Retz extract [97] obtained by sephadex fractionation shows a significant reduction in QS-regulated production of extracellular virulence factors and biofilm formation in *P. aeruginosa*. In turn, electrospray ionisation mass spectrometry revealed significant reduction of 3-oxo-C12-AHL and C4-AHL production in *P. aeruginosa* after treatment with the ellagic-acid-containing fraction. Urolithin A and B can also reduce C6-AHL and 3-oxo-C6-AHL levels and inhibit QS-associated biofilm maturation and swimming motility in *Yersinia enterocolitica*, which contains *yenR* and *yenI* genes (*luxR* and *luxI* orthologs, respectively) [68]. Thus, ellagitannins can realise their anti-QS potential through secondary hydrolysed and metabolised products, which in turn act as AHL synthase inhibitors (Table 1).

Compared to this data, reports about proanthocyanidin anti-QS activity are less numerous. Cranberry-derived A-type proanthocyanidins reduce *P. aeruginosa* swarming motility, an effect that significantly disrupts biofilm formation [98], inhibits the production of QS-regulated virulence determinants and protects *Drosophila melanogaster* from fatal *P. aeruginosa* infection [69]. Monitoring QS signalling gene expression revealed that both LasI/RhlI synthases and LasR/RhlR transcriptional regulators are inhibited. Concomitantly, AHL production quantification using liquid chromatography-mass spectrometry showed reduced AHL production, and proteomics analysis revealed significantly differentially expressed proteins after proanthocyanidin treatment. Molecular docking studies suggest that cranberry-derived A-type proanthocyanidin may bind to QS transcriptional regulators, mainly by interacting with their ligand binding sites. However, this hypothesis (especially in terms of the possibility of such large molecules reaching intracellular targets) should be further tested.

## 4. Conclusions and Future Perspectives

The reviewed data indicate the significant diversity of the chemical structure and bioactivity mechanisms of plant-derived molecules that act as inhibitors of AHL-dependent QS in bacteria.

**Figure 1:**
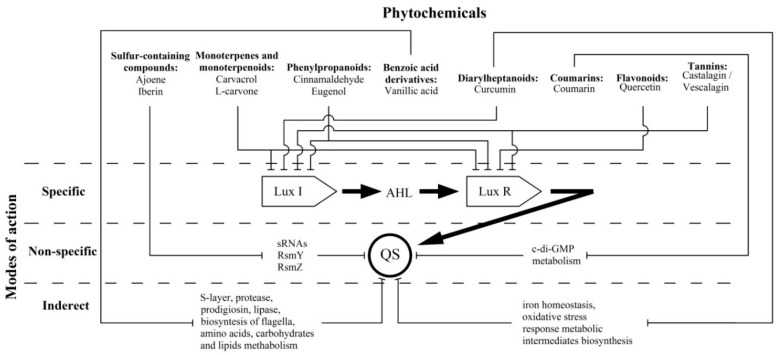
A schematic view of the strategies which phytochemicals use to combat AHL-mediated QS in bacteria.

Most of these compounds (terpenes, phenylpropanoids, flavonoids and tannins) show direct effects on LuxI-type synthases and/or LuxR-type receptor proteins. This fact characterizes their mechanism as “specific” in relation to the discussed QS systems (Figure 1). Thus, the above compounds may be the most attractive “ideal QS inhibitors”, the requirements of which are summarized in a review by Kalia et al. [99]. According to these criteria, terpenes (carvacrol and l-carvone), phenylpropanoids (cinnamaldehyde and eugenol) and flavonoids (including quercetin) are characterized as: (i) highly stable compounds that are resistant to degradation by host metabolism, a factor that allows for their transport to the site of action; (ii) low molecular weight molecules that can penetrate bacterial cells and interact with target proteins; and (iii) high-specificity phytochemicals that directly interact with QS activators. In these terms, polymerised tannins (ellagitannins) do not fully meet the stated requirements due to their relatively high molecular weight, and their ability to be hydrolysed into low-molecular monomers that probably also possess anti-QS activity. Another criterion, namely (iv) no negative influence on host microbiome, is also highly likely for most of the compounds due to the absence of AHL-dependent QS systems in commensal bacterial species that predominantly utilize the AI-2-mediated networks [100]. An exception to this list is phenylpropanoids for which the experimentally evaluated anti-QS spectrum is not limited to AHL-dependent systems, and there is likely a more complex mechanism for their bioactivity than what is now considered. Finally, criterion (v), “no adverse effect on hosts”, is relevant for the discussed phytochemicals that typically possess diverse bioactivity spectra, including some beneficial effects [101], that should be considered too.

Two other referred groups of plant-derived compounds show otherwise modes of action on AHL-dependent quorum sensing which do not interfere with LuxI/LuxR proteins but affected the QS-related intracellular regulatory pathways. These “non-specific” mechanisms were evaluated for sulfur-containing compounds (ajoene and iberin), changing numerous phenotypic traits by lowering the regulatory sRNAs expression, and coumarins leading to QS operons repression by means of c-di-GMP metabolism reduction (Figure 1). Thus, these compounds do not meet specificity criterion (iii), because they have an inhibitory effect on a variety of QS systems, regardless of the nature of the autoinductor and the signal reception [24]. 

Therefore, they may interfere with the normal host microbiome (criterion iv). It should be noted that the practical use of a number of referred plant extracts and their components may be further limited due to the adverse effect on hosts (criterion v). This aspect has been studied partially for a number of compounds [23]; to the greatest extent it is reflected for daily disulfide and other sulfur-containing compounds, the clinical significance of which is limited by toxicity [23].

In turn, vanillic acid (a benzoic acid derivative) and curcumin (from the diarylheptanoids group) exhibit a special version of bioactivity that is related to the alteration of metabolic intermediates involved in QS-dependent virulence factors production. Thus, these compounds mediate “indirect” modes of activity (Figure 1), and they can no longer be considered exclusive QS inhibitors.

Fundamentally, the presented data show the phylogenetically developed variety of anti-infectious strategies relevant in plant health and performed by QS inhibition in phytopathogens [102] that provide new insight into the interactions between plant and bacterial species. At the same time, the presence of stereotypical AHL-mediated QS systems in human and animal pathogens determines the applied aspects of this data as a possibility of transfer of the discussed anti-infection plant-derived compounds in mammalian hosts that can be used as alternatives to antibiotics [8]. In this context, increased understanding of plant-derived molecule modes of action on QS in bacteria should lead to the development of effective QS-disrupting strategies.

Because the whole plant extracts typically contain more than one anti-QS compound and individual constituent phytochemicals are found to be less efficacious than the whole extract [103], a closer look at natural QS inhibitors bioactivity mechanisms may be helpful not only to reconstruct plant-derived molecular mixtures [15] but also for the development of new artificial compositions which exhibit a super-additive effect on quorum sensing. In our opinion, compositions of plant-derived molecules exhibiting a different mode of action are promising, which potentially allow a multiple block on QS-related processes in bacteria. This novel combinatorial approach to QS-disruption will be experimentally verified in our subsequent studies.

## Figures and Tables

**Table 1:** Plant-derived compounds with proved modes of action on AHL-mediated quorum sensing in bacteria.

IUPAC (Trivial) Names of the Compounds Structural Formulas	Plants Source	Anti-QS Effects	Modes of Action	References
**1. Sulfur-Containing Compounds**				
(E)-1-(Prop-2-enyldisulfanyl)-3-prop-2-enylsulfinylprop-1-ene (Ajoene) 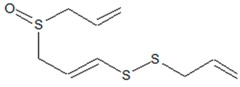	*Allium sativum* L. (garliс)	Inhibition of QS-regulated virulence factors (elastase, rhamnolipid, pyocyanin) and biofilm formation in *P. aeruginosa*; inhibition of *P. aeruginosa* infection in a murine model	Lowering expression of GacA-dependent small regulatory RNAs (RsmY and RsmZ)	[33,37,38]
1-isothiocyanato-3-methylsulfinylpropane (Iberin) 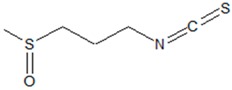	*Armoracia rusticana* G.Gaertn., B.Mey., Scherb. (horseradish), *Brassicaceae* species	Blocking of QS-regulated features, matrix rhamnolypid biosynthesis and biofilm formation in *P. aeruginosa*	Inhibition of GacA-dependent small regulatory RNAs (RsmY and RsmZ); lowering the abundance of the LadS protein (GacS activator)	[34,36,37,38]
**2. Monoterpenes and Monoterpenoids**				
2-methyl-5-propan-2-ylphenol (Carvacrol) 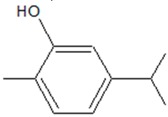	*Origanum vulgare* L. (oregano), Thymus vulgaris L. and other *Lamiaceae* species	Inhibition of QS-regulated violacein biosynthesis and chitinase activity in *C.violaceum*; inhibition of QS-regulated pyocyanin production and biofilm formation in *P. aeruginosa*; lowering of AHL production and QS-controlled genes expression in *Pectobacterium* spp.	Binding to LuxI-type synthase (ExpI) and LuxR-type transcriptional regulator (ExpR)	[39,40,41,42]
2-methyl-5-prop-1-en-2-ylcyclohex-2-en-1-one (l-carvone) 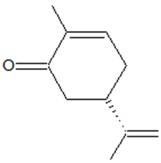	*Carum carvi* L. (caraway), *Mentha spicata* L. (spearmint)	Inhibition of QS-regulated swinging/swarming motility and reduction of AHL production in *H.alvei*	Binding to LuxI-type synthase (HalI) and LuxR-type transcriptional regulator (HalR)	[43]
**3. Phenylpropanoids**				
(2*E*)-3-Phenylprop-2-enal (Cinnamaldehyde) 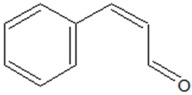	*Cinnamomum* species, *Hyacinthus orientalis* L. (chinese cassia), *Pogostemon cablin* Benth. (patchouli)	Inhibition of QS-regulated virulence factors (protease, elastase, pyocyanin) and biofilm formation in *P. aeruginosa*; inhibition of QS-controlled extracellular protease, /swarming and biofilm formation in *P. fluorescens*; lowering of AHL production; pleiotropic effects on AI-2 mediated QS	Binding to LuxR-type receptor proteins and/or interaction with LuxI-type synthase (LasI)	[28,44,45,46]
2-Methoxy-4-(prop-2-en-1-yl)phenol (Eugenol) 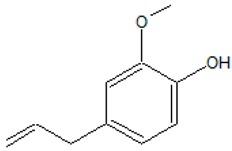	*Syzygium aromaticum* L., *Myrtales* species	Inhibitory effects on QS-biosensors; inhibition of QS-regulated elastase, protease, pyocyanin and pyoverdine biosynthesis in *P. aeruginosa*; inhibition of biofilm formation, matrix polysaccharides and rhamnolipid production;lowering of QS signaling molecules and QS-controlled genes expression in *Pectobacterium* spp.;pleiotropic effects on AI-2 mediated QS	Binding to LuxR-type proteins (LasR and ExpR); down-regulation of AHL synthases genes (*lasI* and *rhlI*); interaction with LuxI-type synthase (ExpI)	[47,48,49]
**4. Benzoic Acid Derivatives**				
4-hydroxy-3-methoxybenzoic acid (Vanillic acid) 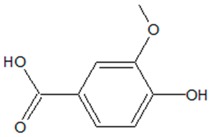	*Actinidia deliciosa* (A.Chev.) C.F.Liang, A.R.Ferguson (kiwifruit)	Inhibition of QS-regulated virulence and biofilm formation in *S. marcescens*; increased survival of *C. elegans* upon *S. marcescens* infection	Changing the proteins content involved in S-layers, protease, prodigiosin and lipase production, targeting flagella, amino acids, carbohydrates and fatty acids biosynthesis	[50,51]
**5. Diarylheptanoids**				
(1E,6E)-1,7-Bis(4-hydroxy-3-methoxyphenyl)hepta-1,6-diene-3,5-dione (Curcumin) 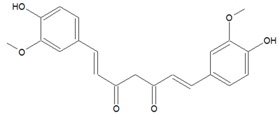	*Curcuma longa L*. (turmeric)	Inhibition of QS-regulated violacein biosynthesis in *C. violaceum* and virulence factors production in *Vibrio* sp. and *S. marcescens*; inhibition of pyocyanin biosynthesis, elastase/protease activity, and biofilm formation in *P. aeruginosa*; reduction *P. aeruginosa* pathogenicity in *C. elegans* infection models; lowering AHL production in *P. aeruginosa* and *A. sobria*	Interaction with LuxI-type synthases; down-regulation of LuxI-type and LuxR-type proteins genes (*lasI*, *lasR*, *rhlI* and *rhlR*); targeting iron homeostasis, oxidative stress response and biosynthesis of metabolic intermediates involved in virulence factors production	[52,53,54,55]
**6. Coumarins**				
2*H*-chromen-2-one (Coumarin) 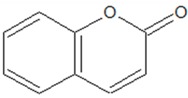	*Dipteryx odorata* (Aubl.) Willd. (tonka bean), *Anthoxanthum odoratum* L., *Galium odoratum* L., *Hierochloe odorata* L., *Cinnamomum verum* J.Presl and *Melilotus officinalis* L.	Inhibitory effects on QS-biosensors; inhibition of violacein biosynthesis in *C. violaceum*; inhibition of QS-regulated, phenazines production, swarming motility and biofilm formation in *P. aeruginosa*	Reduction of c-di-GMP metabolism	[56,57,58,59,60,61]
**7. Flavonoids**				
2-(3,4-dihydroxyphenyl)-3,5,7-trihydroxy-4*H*-chromen-4-one (Quercetin) 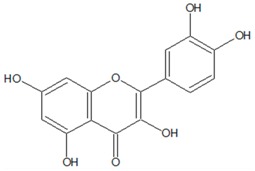	*Allium cepa* L. (red onion), *Brassica oleracea* L. (leaf cabbage or “kale”), many others fruits, vegetables, leaves, and grains	Inhibition of QS-regulated bioluminescence in *V. harveyi*; Inhibition of violacein production in *C. violaceum*; Inhibition of pyocyanin production, proteolytic, elastolytic activities, swarming motility and biofilm formation in *P. aeruginosa*	Binding to LuxR-type receptor proteins (LasR, RhlR and CviR)	[62,63,64,65]
**8. Tannins**				
(46*R*)-7,8,9,12,13,14,25,26,27,30, 31,32,35,36,37,46-hexadecahydroxy-3,18,21,41,43-pentaoxanonacyclo[27.13.3.1^38,42^.0^2,20^.0^5,10^.0^11,16^.0^23,28^.0^33,45^.0^34,39^]hexatetraconta-5,7,9,11,13,15,23,25,27,29(45),30,32,34(39),35,37-pentadecaene-4,17,22,40,44-pentone(Castalagin: R1=OH, R2=H/Vescalagin: R1=H, R2=OH) 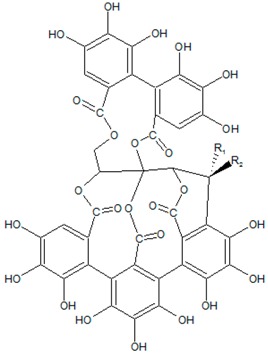	*Conocarpus erectus* L, *Anogeissus leiocarpa* (DC.) Guill. & Perr., *Terminalia avicennioides* Guill., Perr. Fl. Seneg. Tent.	Inhibition of QS-controlled genes and QS-regulated virulence factors in *P. aeruginosa*; lowering of AHL production; increased survival of *C. elegans* upon *P. aeruginosa* infection	Interaction with QS-related proteins (including LuxI-type syntheses) via precipitation mechanism; bioactivity may be explained by secondary hydrolyzed and metabolized products	[45,66,67,68,69]
